# A meta-analysis for prevalence of infectious laryngotracheitis in chickens in mainland China in 1981–2022

**DOI:** 10.1186/s12917-024-03991-3

**Published:** 2024-04-09

**Authors:** Xingping Hong, Huiling Zhang, Xiaorong Zhang, Xue Ping Zhang, Tangjie Zhang

**Affiliations:** 1 Institute of Comparative Medicine, College of Veterinary Medicine, Yangzhou University, Yangzhou, Jiangsu 225009 China; 2 Jiangsu Co-innovation Center for Prevention and Control of Important Animal Infectious Diseases and Zoonoses, Yangzhou, Jiangsu 225009 China; 3 Independent Person, New York, NY 11355 USA

**Keywords:** Infectious laryngotracheitis, Infectious laryngotracheitis virus, Chicken, Meta-analysis, Prevalence rate

## Abstract

**Background:**

Infectious laryngotracheitis (ILT) is a highly infectious upper respiratory tract disease of chickens caused by infectious laryngotracheitis virus or Gallid herpesvirus 1 (GaHV-1). ILT is an important respiratory disease of chickens and annually causes significant economic losses in the chicken industry. Although numerous relevant studies have been published, the overall prevalence of ILT infection among chicken in mainland China is still unknown, and associated risk factors need to be evaluated to establish preventive measures.

**Results:**

The present study reviewed the literature on the prevalence of ILT in chickens in China as of December 20, 2022, retrieved from six databases—CNKI, Wanfang, VIP, PubMed, Web of Science, and ScienceDirect—were used to retrieve relevant studies published between January 1, 1981 and December 20, 2022. The literature quality of studies was assessed, and 20 studies with a total of 108,587 samples were included in the meta-analysis. Results of the meta-analysis showed that the overall prevalence of ILT was 10% (95% confidence interval: 8 –12%) through the random-effects model, which showed high heterogeneity, I^2^ = 99.4%. Further subgroup analyses showed that the prevalence of ILT decreased over time; furthermore, the prevalence in Northwest China was slightly lower than that in North China and South China, and the prevalence estimated using the diagnostic technique AGP was higher than that reported using other diagnostic techniques.

**Conclusions:**

ILT is prevalent to some extent in mainland China. Given that the ILT attenuated live vaccine has a certain level of virulence and the prevalence differences between regions, we recommend controlling breeding density, improving immunization programs and continuously monitoring viruses and to prevent ILT prevailing in mainland China.

## Background

Infectious laryngotracheitis (ILT) is a highly infectious upper respiratory tract disease of chickens caused by infectious laryngotracheitis virus (ILTV) or Gallid herpesvirus 1 [GaHV-1]), which is naturally transmitted via the upper respiratory tract, ocular route, or oral route [1]. Chickens of all ages are affected, but chickens over 3 weeks of age are most susceptible to infection [2], characterized by dyspnea, coughing up of blood-containing exudates, swelling of throat and organ mucosa, bleeding, and erosion. The disease was first reported in Canada in 1925, then in the United States in 1926, in Australia and the United Kingdom in 1935, and in Europe in 1940 [3]. Since its first detection in China, the disease in 1959 in Guiyang City [4], ILT has been reported in several places in the country. With an infection rate of approximately 90% [4], ILT spreads rapidly in a chicken population, causing deaths; the consequent decrease in chicken and egg production severely affects the poultry industry [5]. At present, in addition to conventional diagnostic methods, including isolation and identification of ILTV, serological testing, advanced biotechnology tools such as PCR, quantitative real-time PCR, next-generation sequencing, etc. are being used for accurate diagnosis and epidemiological research of ILTV.

Although there are numerous relevant studies on chicken ILT, many published studies about the prevalence only focus on a province or certain areas, while the overall prevalence of ILT infection among chicken is still unknown in mainland China [6–8]. A specific transformation is usually applied to each study’s proportion estimate for better approximation to the normal distribution [9], as required by the assumptions of conventional meta-analysis models and then the meta-analysis was performed on the transformed scale to evaluate the pooled prevalence of ILT among chicken in mainland China and to analyze the associated risk factors for ILT to provide helpful information for preventing and controlling ILT.

## Results

### Literature search results

**Fig. 1:**
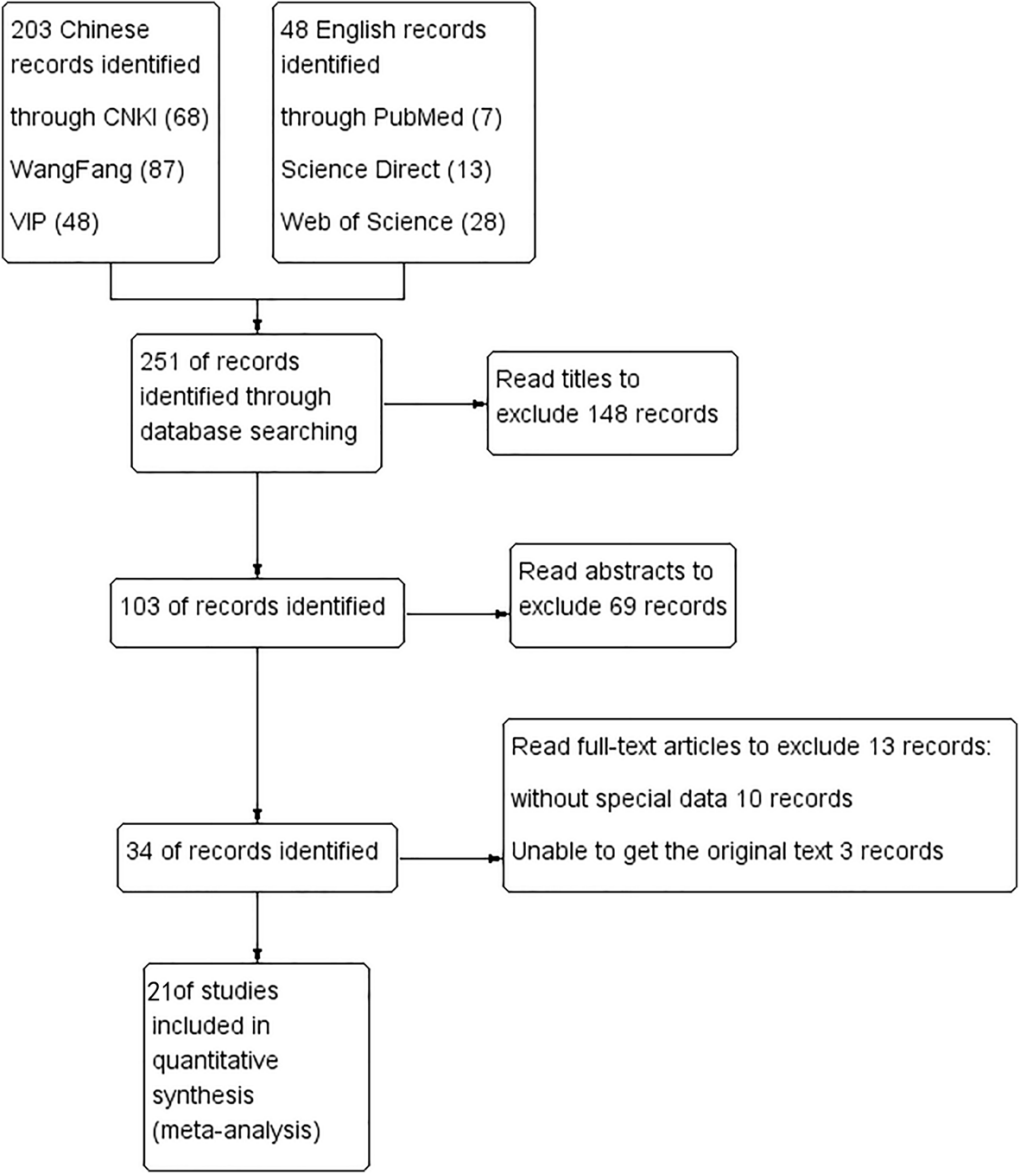
Flow diagram of screening of epidemiological studies on infectious laryngotracheitis (ILT)

Using the search strategy described above, 251 studies were retrieved, which were screened on the basis of the method recommended by the Cochrane manual. The title, abstract, and full text of the studies were reviewed, respectively, and some studies were excluded at each step on the basis of the exclusion criteria; finally, 21 studies were included for quality evaluation [6–8, 10–27]. The screening procedure and outcomes are depicted in Fig. 1.

### Quality evaluation and data extraction

**Fig. 2:**
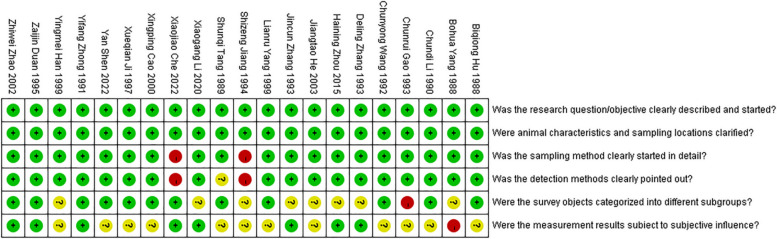
Quality assessment of screened studies on infectious laryngotracheitis (ILT)

The quality of each study was evaluated; the corresponding scores are depicted in Fig. 2. Green represents 2 points, indicating low risk; yellow represents 1 point, indicating uncertainty; and red represents 0 points, indicating high risk. During quality evaluation, the study by Jiang et al. [15] was ascribed less than 7 points; thus, it was excluded, and the other 20 studies were included in the statistical analysis. Except for 1 article which used fluorescence quantitative PCR as the diagnostic method [11], and all the other methods were serological tests. The literatures involved in this study were only from unvaccinated ILT chickens. The following data were extracted from these 20 studies: the first author and the publication year of the journal, sampling area, sampling time, diagnostic technique, incidence, and the total number of samples (Table 1).

**Table 1:** Baseline data of the studies included in the meta-analysis

Author	Sampling year	Sampling area	Diagnostic techniques	Positive samples/Total samples	Detection rate (95% CI)	Quality score
Xiaojiao Che	2019	Gansu	Unclear	523/10411	0.05(0.05,0.05)	8
Xiaojiao Che	2018	Gansu	Unclear	3404/42052	0.08(0.08,0.08)	8
Xiaojiao Che	2017	Gansu	Unclear	910/40643	0.02(0.02,0.02)	8
Yan Shen	2021	Anhui	PCR	5/472	0.01(0.00,0.02)	11
Haining Zhou	2015	Ninxia	ELISA	24/450	0.05(0.03,0.07)	11
Xiaogang Li	2009	Henan	AGP	240/1224	0.20(0.17,0.22)	11
Jiangtao He	2000	Xinjiang	AGP	2/115	0.02(−0.01,0.04)	10
Zhiwei Zhao	2001	Henan	AGP	77/725	0.11(0.08,0.13)	12
Xingping Cao	2000	Yunnan	AGP	30/387	0.08(0.05,0.10)	11
Lianru Yang	1998	Neimenggu	AGP	626/1612	0.39(0.36,0.41)	11
Yingmei Han	1998	Gansu	AGP	30/290	0.10(0.07,0.14)	10
Xueqian Ji	1992	Gansu	AGP	1/592	0.00(−0.00,0.00)	11
Delin Zhang	1990–1992	Gansu	AGP	446/4988	0.09(0.08,0.10)	11
Chunrui Gao	1990	Neimenggu	AGP	0/26	0.04(−0.04,0.11)	9
Chunyong Wang	1990	Gansu	AGP	20/732	0.03(0.02,0.04)	11
Jincun Zhang	1989–1990	Gansu	AGP	10/363	0.03(0.01,0.04)	11
Biqiong Hu	1987	Ninxia	AGP	22/305	0.07(0.04,0.10)	11
Chundi Li	1986	Yunnan	RIHA	16/131	0.12(0.07,0.18)	11
Bohua Yang	1986	Jiangsu	SPA	221/845	0.26(0.23,0.29)	9
Yifang Zhong	1984–1988	Xinjiang	AGP	122/917	0.13(0.11,0.16)	12
Shunqi Tang	1989	Jiangsu	AGP	53/895	0.06(0.04,0.07)	10
Zaijin Duan	1995	Jiangxi	AGP	118/412	0.29(0.24,0.33)	12

### Heterogeneity analyses

**Fig. 3:**
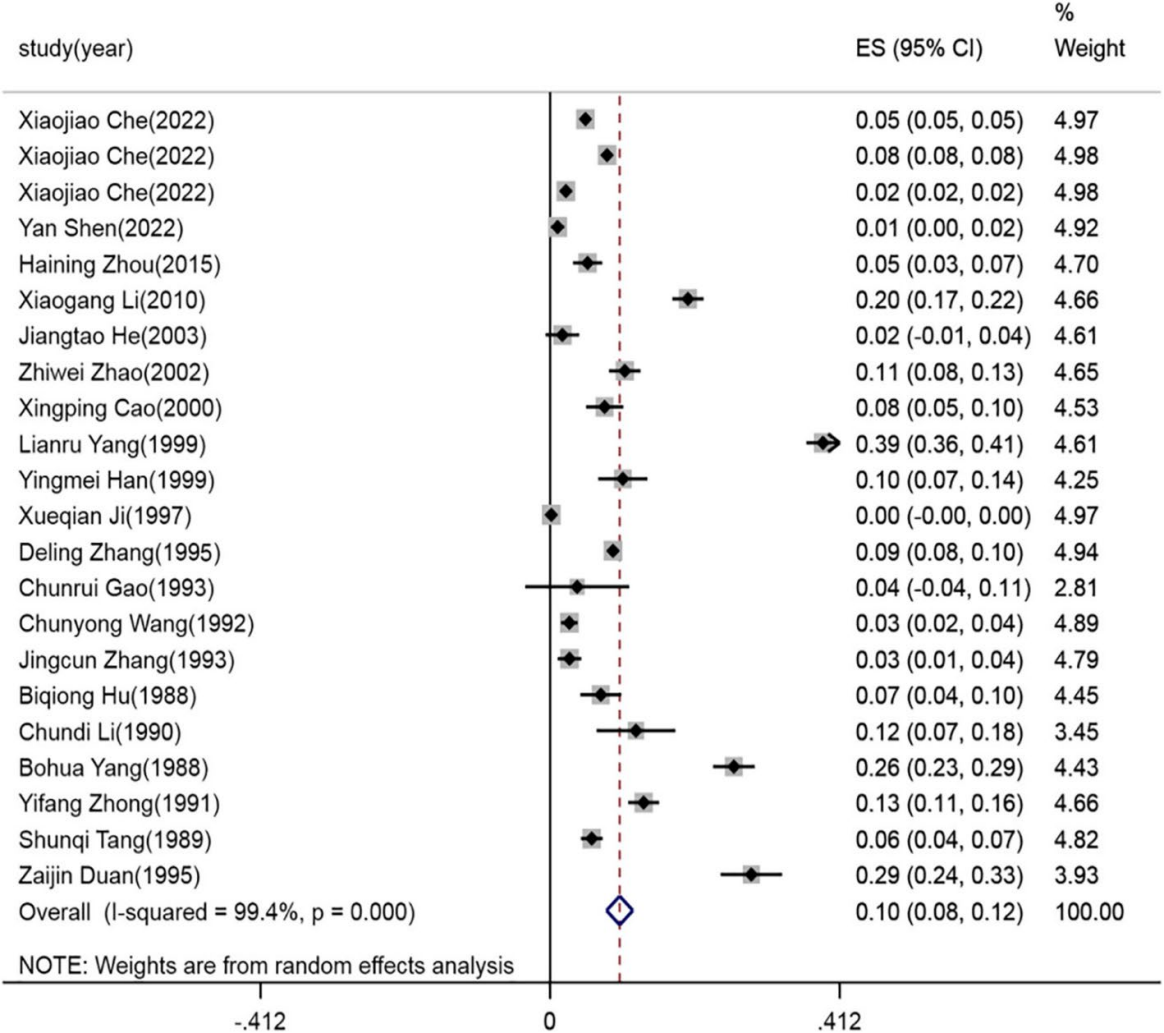
Summary forest plot of the estimated ILT incidence random effects analyses; ES: Effect size (positive proportion)

Double arcsine conversion was applied to the ILT prevalence data reported in the 20 studies, respectively, to estimate the prevalence of ILT and 95% CI. The heterogeneity index (I^2^) was 99.4% (*p* < 0.001), indicating that the 20 studies included in this meta-analysis were highly heterogeneous; therefore, the random effects model was applied. Results of the meta-analysis showed that the incidence of ILT was 10% (95% CI: 8 –12%), as shown in Fig. 3.

**Fig. 4:**
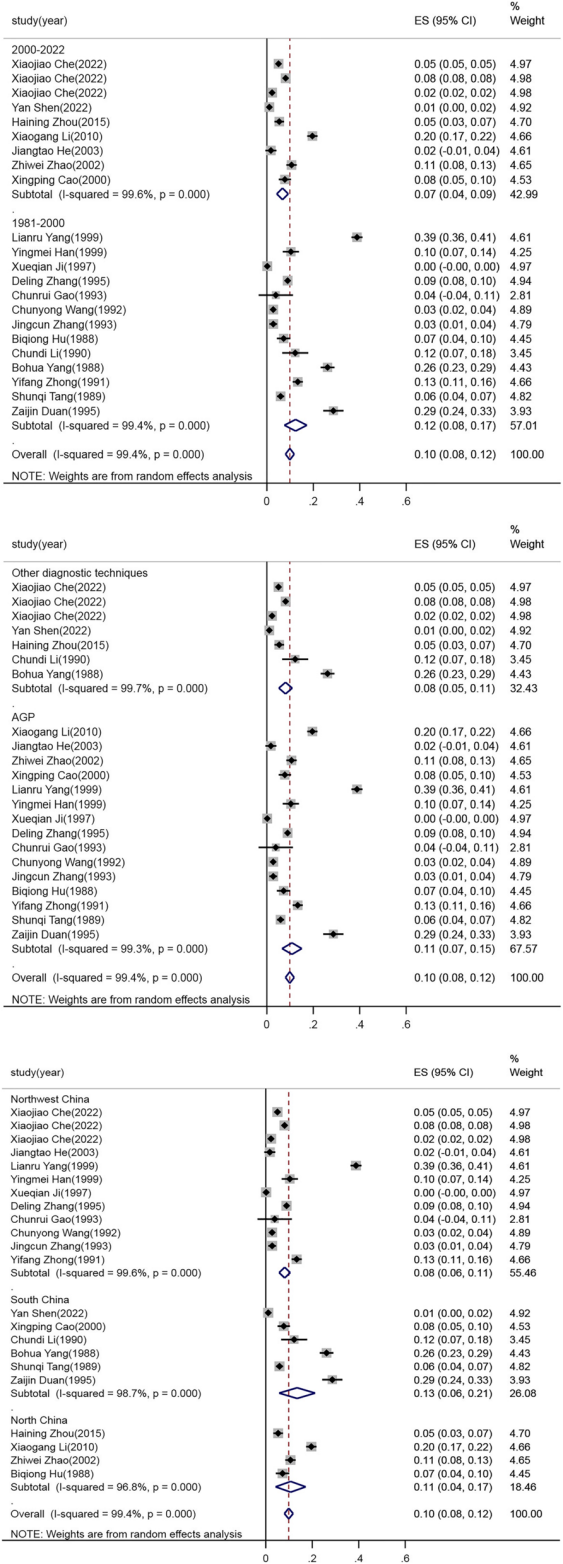
Forest plot of the ILT estimated incidence subgroup with random effects analyses. Upper: the subgroup analysis of different years；Intermediate: the subgroup analysis of different diagnostic techniques; Lower: the subgroup analysis of different areas. ES: Effect size (positive proportion)

The possible causes of morbidity heterogeneity were analyzed. The studies were divided into subgroups on the basis of study period (before and after 2000), diagnostic technique (AGP and two other subgroups), and sampling area (Northwest China, South China, and North China), and heterogeneity data presented in Table 2. The subgroup analysis according to study period showed that the incidence rate was 12% (95% CI: 8 –17%) during 1988–1999 and 7% (95% CI: 4 –9%) during 2000–2022, with an overall decreasing trend over time, as shown in Fig. 4(Upper). The subgroup analysis according to diagnostic technique showed that the incidence rate was 11% (95% CI: 7 –15%) in the AGP subgroup, and 8% (95% CI: 5 –11%) in the other subgroup, as shown in Fig. 4 (Intermediate). The incidence rate was 8% (95% CI: 6 –11%) in Northwest China, 13% (95% CI: 6 –21%) in South China, and 11% (95% CI: 4 –17%) in North China, as shown in Fig. 4 (Lower).

**Table 2:** The subgroup analysis and heterogeneous data of the prevalence of infectious laryngotracheitis in chickens in mainland China

Variables	Numbers of studies	Numbers of samples	Numbers of positive samples	Prevalence rate(95%CI)	Heterogeneity		
					X^2^	*P*-value	I^2^/%
**Region**				0.10(0.08,0.12)	3705.39	0.000	99.4
Northwest China	12	102,741	6095	0.08(0.06,0.11)	3052.91	0.000	99.6
South China	6	3142	443	0.13(0.06,0.21)	390.20	0.000	98.7
North China	4	2704	363	0.11(0.04,0.17)	92.75	0.000	96.8
**Diagnostic techniques**				0.10(0.08,0.12)	3705.39	0.000	99.4
Other diagnostic techniques	7	95,004	5103	0.08(0.05,0.11)	1789.34	0.000	99.7
AGP	15	13,583	1798	0.11(0.07,0.15)	1896.65	0.000	99.3
**Sampling times**				0.10(0.08,0.12)	3705.39	0.000	99.4
1981–2000	13	12,108	1686	0.12(0.08,0.17)	1869.17	0.000	99.4
2000–2023	9	96,479	5215	0.07(0.04,0.09)	1803.88	0.000	99.6

### Bias and sensitivity analysis

**Fig. 5:**
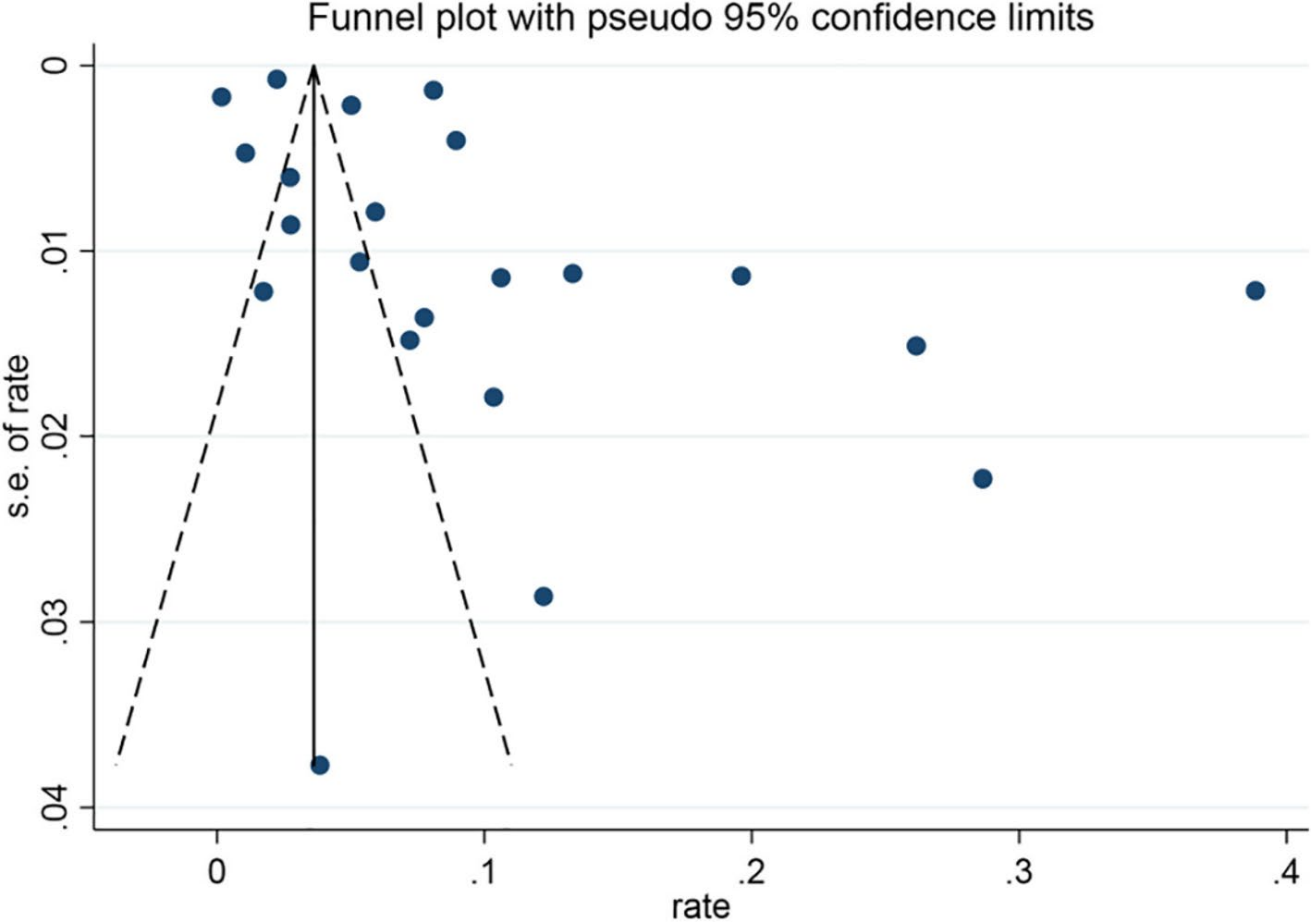
Funnel plot of the meta-analysis of epidemiological studies on infectious laryngotracheitis (ILT); rate: positive rate; s.e: Standard error of positive rate

A funnel plot was used to assess whether there was publication bias in the included studies (Fig. 5). The distinct asymmetry in the funnel plot (studies represented by dots) indicated significant publication bias in the included studies.

**Fig. 6:**
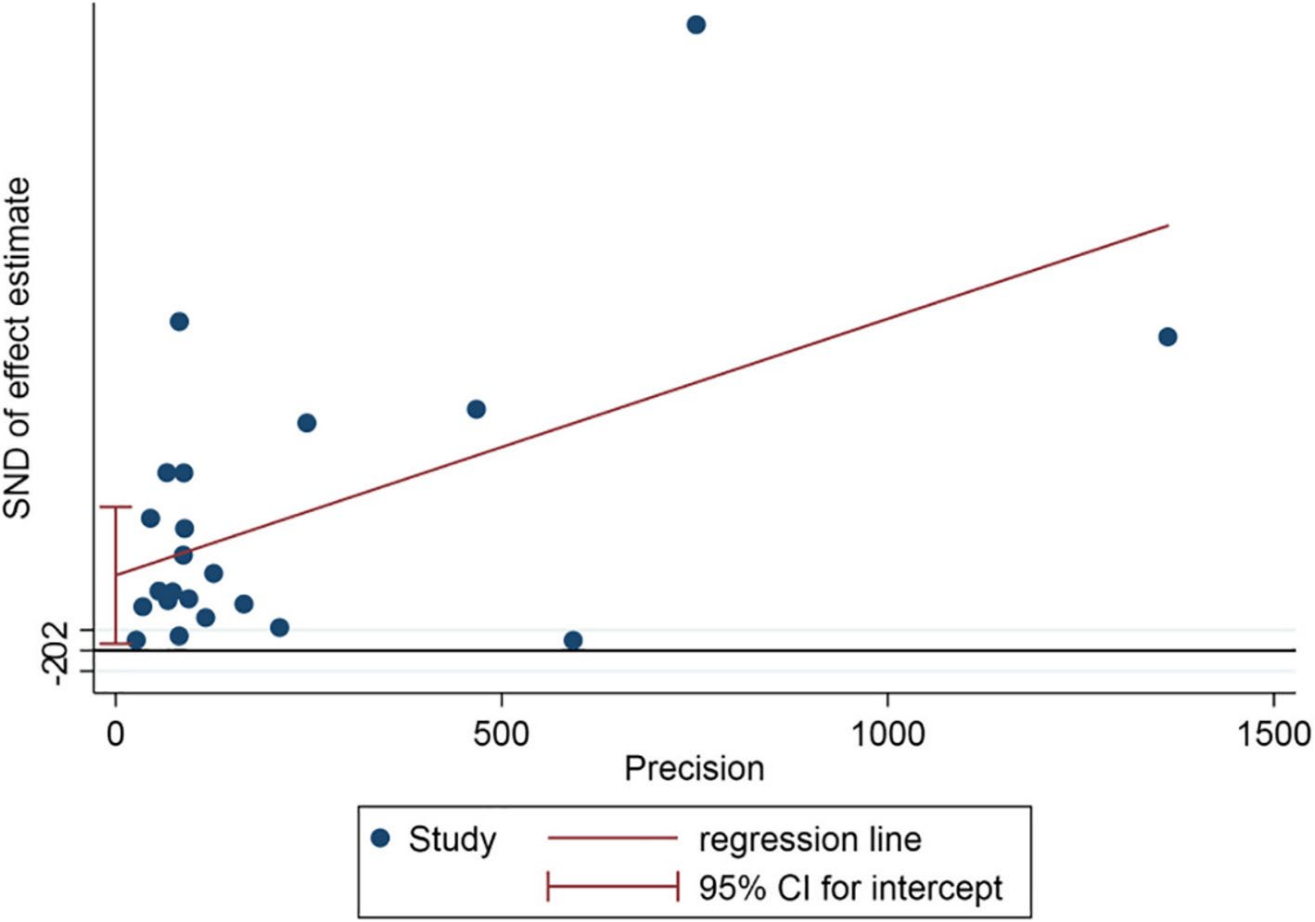
Egger test of publication bias among epidemiological studies on infectious laryngotracheitis (ILT); SND: Standard normal deviate; Precision: Inverse of the variance

The Egger test was used to further analyze the publication bias in the included studies, and the results indicated significant publication bias in the included studies (*p* = 0.033 and *p* < 0.05; Fig. 6) [28, 29].

**Fig. 7:**
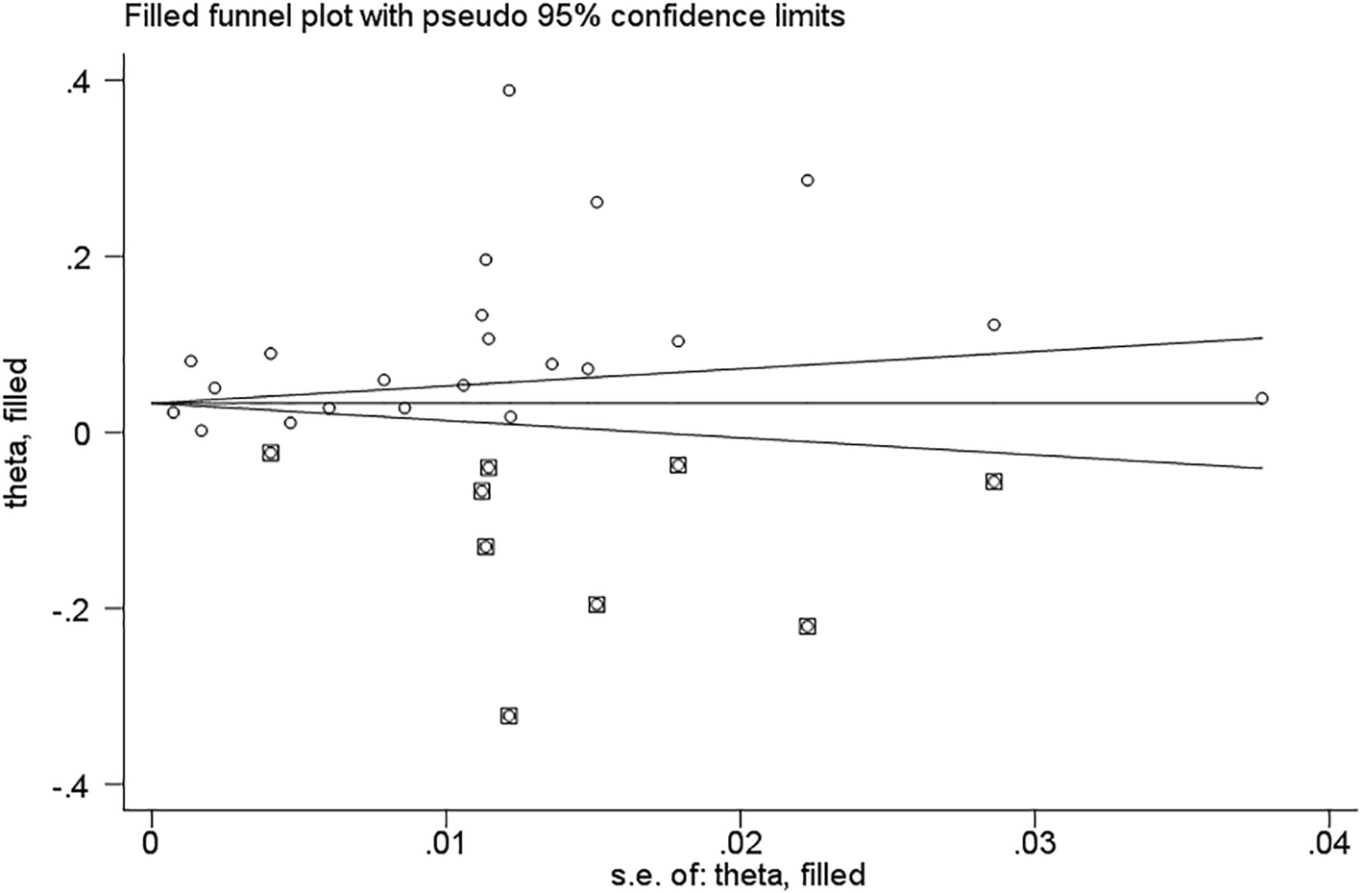
Filled funnel plot of metaanalysis with “trim-and-fill” method 〇 indicated observed studies ⌼ indicated missed studies

Based on the assumption that publication bias leads to funnel graph asymmetry, an iterative method is used to estimate the number of missing studies, and a new meta-analysis is conducted using trim and filling method (Metatrim command from Stata software) to determine the impact of publication bias on research results [28, 30]. After including nine additional virtual studies, meta-analysis was performed again, the adjusted *p* = 0.000, (Fig. 7). The heterogeneity was still significant, indicating that the corrected results were the combined results were robust.

**Fig. 8:**
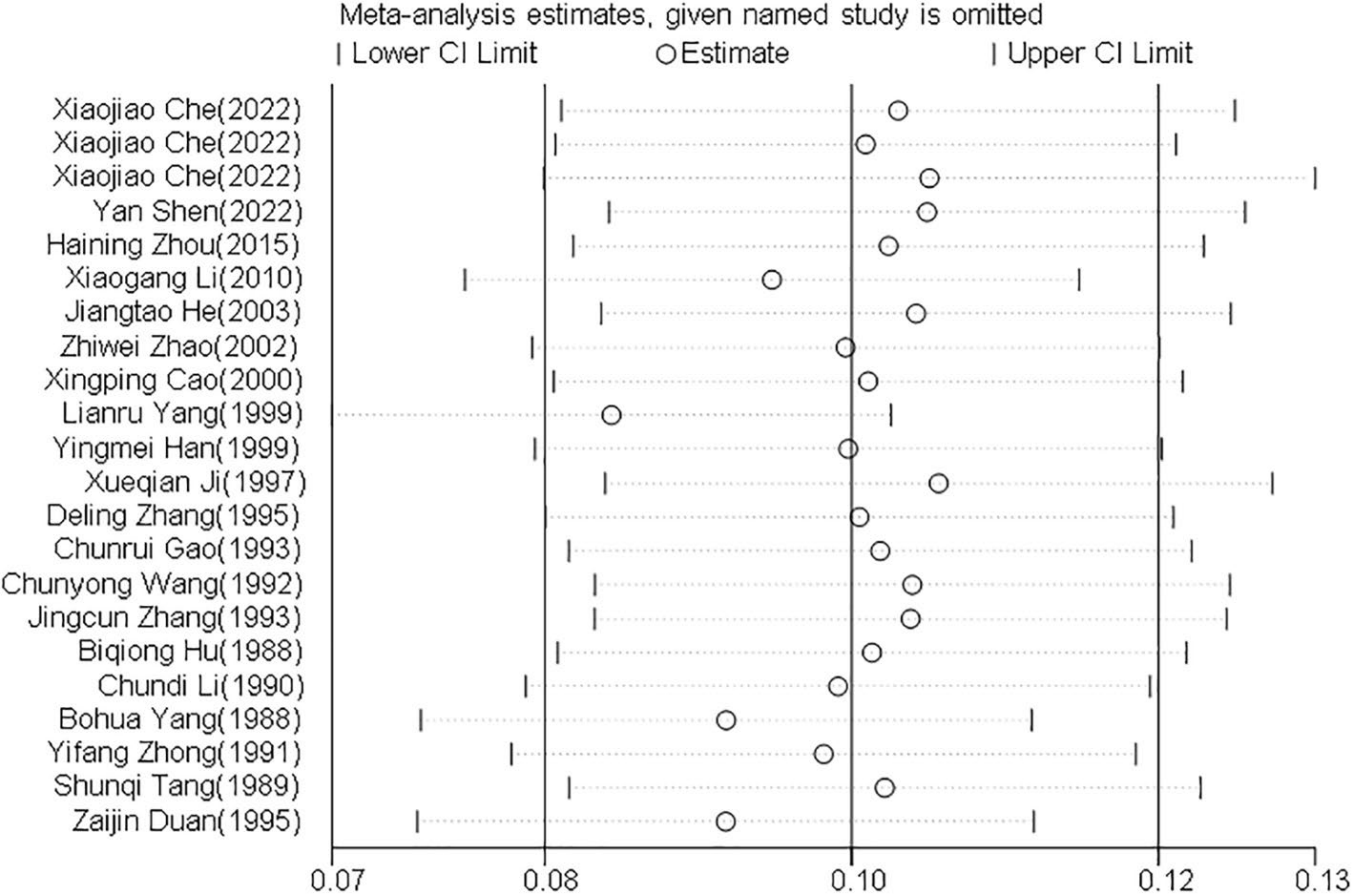
Sensitivity analysis of epidemiological studies on infectious laryngotracheitis (ILT)

The results of the sensitivity analysis showed that the incidence ratio of ILT was within 95% CI (Fig. 8), indicating that the results of the meta-analysis would not change significantly with the change in the number of studies, indicating robustness.

## Discussion

Meta-analysis is an approach to formally, systematically and quantitatively analyze multiple existing research studies with the same research purpose, and to synthesize new research findings based upon the existing data, in order to increase sample size and improve statistical testing efficiency. A key application of meta-analysis is the pooling of proportions, such as prevalence of a specific disease or infection [31]. The Freeman-Tukey double-arcsine transformation, are popular tools for better approximating to the normal distribution and stabilizing the variance of each study’s proportion in meta-analysis methods [32–34]. Here we carried out the first systematic review and meta-analysis based on the pooled prevalence of ILT among chicken over the past 42 years in China and analyzed the associated risk factors for ILT. Based on the results, the prevalence of ILT was 10% through the random-effects model, this finding is lower than the 20% prevalence in Nigeria [35], the 17.33% prevalence in Bangladesh [36] and the 19.4% prevalence in Ethiopian [37], all of which were based on investigations of commercial poultry production systems. The prevalence of ILT may vary depending on factors such as region, breeding conditions, and bird management. The epidemic dynamics of ILT are also related to seasonal changes. The variation in the prevalence rates of ILT in different regions could be attributed to the system of rearing, stocking density and the use of live vaccines. For example, some studies reported that the prevalence was significantly higher in backyard chickens than commercial production systems. The overall prevalence for ILT could range from 28% ~ 77% in backyard poultry production systems [37–39].

Heterogeneity is an important consideration in systematic reviews, as high heterogeneity may imply that it is not suitable to perform meta-analysis. Methods of assessing heterogeneity are calculating a statistical test for heterogeneity (the I value), visual evaluations of forest plots, conducting meta-regression or subgroup analysis. In multiple meta-regressions, more predictors are used in the same meta-regression model. Subgroup analyses are a special form of meta-regression. As mentioned earlier, there was highly heterogeneous in this meta-analysis. But there were only three variables, so subgroup analysis was used. In the subgroup analysis, when the included studies were classified into two subgroups according to the study period—before and after 2000—the incidence of ILT showed a decreasing trend over time, which may be attributed to strict biosafety measures and scientific breeding to enhance the control of ILT. China’s poultry industry is going through rapid industrialization, characterized by intensification of farming, horizontal consolidation and vertical integration during the past four decades. The previous model of free-range chicken farming by farmers, similar to backyard chickens mentioned above, that lacking strict biosafety measures has gradually shifted towards large-scale, intensive, and modern poultry industry, which have a complete set of strict biosafety measures [40–42] and scientific management measures.

The detection methods for ILT mainly included serological methods such as AGP and ELISA for antibody detection, and PCR for viral nucleic acid detection. There is only one serotype of ILTV, for which the clinical application of PCR takes a long time, while the serological testing is easy to promote and more common used at the grassroots in mainland China. Serological tests in this study were performed on non-immunized chicken, a positive serological result indicated that the chicken was infected or currently suffering ILT. Most studies included in the present meta-analysis used the serological diagnostic technique AGP for antibody detection; it has been reported that AGP shows lower sensitivity for the detection and identification of ILTV than other methods such as ELISA and PCR [43, 44]. Prevalence of ILT estimated by using AGP was higher than that estimated by other methods. Because AGP is a serological antibody detection test, vaccine-immunized chickens with antibodies would also test positive, resulting in a higher the detection rate than that of PCR and other diagnostic techniques.

Publication bias is a serious issue in systematic reviews and meta-analyses, which can affect the validity and conclusions. Currently, approaches to dealing with publication bias can be divided into two classes: selection models and funnel-plot-based methods. Selection models use weight functions to adjust the overall effect size estimate and are usually employed as sensitivity analyses to assess the potential impact of publication bias. Funnel-plot-based methods include examination of a funnel plot (Egger’s test), regression, and the trim and fill method. The results of the trim and fill method and the sensitivity analysis indicated that the results of the meta-analysis were robustness. Combined with the Egger’s test, it is shown that publication bias has no significant impact on the results, and our results are validity.

Rapid expansion of poultry populations has led to an increase in ILT outbreaks in many poultry-producing areas, particularly in areas with high poultry density. The subgroup analysis according to study area showed that the prevalence of ILT was the highest in South China, followed by that in North China, and slightly lower in Northwest China than. This finding may be explained to the fact that the key chicken-raising provinces are distributed in East China and North China, and the higher incidence of ILT is higher in North and South China owing to the higher breeding density and higher demand.

The literature reviewed in this article is based on tests of chickens that have not been vaccinated against ILT，but studies have shown that the current incidence of the disease is partially attributable to the use of live-attenuated ILT vaccines, which have been widely used since their introduction in the mid-twentieth century. Vaccination effectively prevents ILTV infection [45]. Current (attenuated live) ILT vaccines can offer good protection, however, the strains of ILTV used in vaccines may lead to latent infections [3, 46]. These potential carriers are the source of transmission of the virus to unvaccinated chickens [47]. When chickens receive live-attenuated vaccines, the ability of the virus to regain virulence and spread increases the prevalence of ILT. Inadequately vaccinated chicken populations at a farm may be exposed to ILTV following the introduction of vaccinated young chickens. According to studies in Taiwan [48] and Australia [43], live-attenuated vaccines have replaced wild-type viruses as the cause of ILT outbreaks in these regions. So rigorous on-site biosafety is crucial for ILT disease control, including unvaccinated chickens cannot be mixed with vaccinated chickens. In this study, Zhang Deling [23] also conducted serological testing on both immunized and non-immunized chicken herds. Their results showed that the prevalence of ILT in non-immunized chicken herds was 8.9%, while the prevalence of ILT in immunized chicken herds was 28.3%, indicating that the utilization of ILT vaccine could lead to an increase in seroconversion of ILT. Considering the latent infections associated with attenuated live vaccines, it is recommended not to vaccinate the ILT vaccine unless there is an ILT present in the field. Or at least the vaccination protocols should be modified.

The present study has certain limitations: 1) The time span for inclusion in the study is long, but the small number of studies included in this analysis.. 2) inclusion of few epidemiological studies. 3) Some studies had a small sample size that may have affected the overall prevalence.

## Conclusion

In conclusion, the present study investigated the overall prevalence of ILT in China through systematic review and meta-analysis. The prevalence of ILT decreased over time and that the prevalence in Northwest China was slightly lower than that in other regions. We suggest that controlling breeding density, improving immunization programs and continuously monitoring viruses to prevent the prevalence of ILT in mainland China.

## Materials and methods

### Search strategy

The study was conducted according to the Preferred Reporting Items for Systematic Reviews and Meta-Analyses (PRISMA) guidelines. The PRISMA checklist was used to ensure the inclusion of all relevant information in the analysis. Six key databases—China National Knowledge Network (CNKI), Wanfang, VIP, PubMed, Web of Science, and ScienceDirect—were used to search the existing literature on the prevalence of ILT in China from January 1, 1981 to December 20, 2022, and languages were limited to Chinese and English.

Literature search was performed using a combination of subject-specific terms and free-text terms. The Chinese search terms were “infectious laryngotracheitis” or “ILT” and “prevalence” or “survey.” The English search terms were “ILT” or “infectious laryngotracheitis” and “epidemiological” or “survey” and “China.”

### Inclusion and exclusion criteria

Full-texts articles were obtained, and studies were included in the review if they met the following criteria: (i) studies performed in China, (ii) studies performed in chickens, (iii) cross-sectional studies, (iv) the studies on the etiological or serological investigation of ILT, and (v) studies with more than 20 clinical samples of ILT with specific data.

The exclusion criteria were as follows: (i) studies investigating suspected cases, (ii) studies on non-chicken avian species (e.g., ducks, birds, and turkeys), (iii) republished articles, and (iv) studies with less than 20 samples, and (v) literature reviews.

Literature review was performed independently by two reviewers. If the results were inconsistent, the discrepancies were resolved by a third party or through discussion and negotiation. Articles that did not meet the inclusion criteria were excluded. The screened literature for individual chicken prevalence was carefully read, and the following information was collected: author and publication year, sampling time and sampling location, the total number of samples collected and the number of positive samples, and diagnostic methods. Authors were contacted by email if any data were missing.

### Literature quality assessment

Cross-sectional studies in animals differ from randomized clinical trials; their systematic evaluation methods are not mature, and there is no control group in such studies. Therefore, the systematic evaluation methods of animal cross-sectional clinical trials were adjusted on the basis of Cochrane quality evaluation, and the study quality was assessed by evaluating the following six items using RevMan 5.4. Scores were assigned on the basis of a simple scale: 2 for “yes,” 1 for “not sure,” and 0 for “no.” ① Are the research questions/objectives clearly described and stated? ② Are animal characteristics and sampling sites clarified? ③ Is the sampling method explained in detail? ④ Is the virus detection method clearly indicated? ⑤ Are the respondents divided into different subgroups? ⑥ Is the measurement outcome subject to subjective influence?

The review and identification of the literature were carried out independently by two researchers, and if the results were inconsistent, the discrepancies were resolved by a third party through discussion or negotiation. Studies for which the total quality evaluation value was lower than 7 points were excluded from statistical analysis.

### Statistical and heterogeneity analyses

Stata software (version 15) was used for meta-analysis, and the double-arcsine transformation (PFT) method was used to bring the data closer to normal distribution [9]. The PFT conversion formula was as follows:

$$\textrm{t}=\textrm{asin}\ \left(\textrm{sqrt}\ \left(\textrm{r}/\left(\textrm{n}+1\right)\right)+\textrm{asin}\ \left(\textrm{sqrt}\ \left(\textrm{r}+1\right)/\left(\textrm{n}+1\right)\right)\right)$$

$$\textrm{set}=\textrm{sqrt}\ \left(1/\left(\textrm{n}+1\right)\right)$$

$$p=\mathit{\sin}\ \left(t/2\right)\hat{\phantom{0}}2$$

where t is the detection rate after conversion, n is the total number of samples, r is the number of positive samples, set is standard error, and *p* is the final detection rate.

The *p* value and I^2^ value were further used to evaluate the heterogeneity between studies, and the appropriate effect model was selected to express the effect size with 95% confidence interval (CI). When *p* ≥ 0.1 and I^2^ < 50%, it was considered that there was no statistical heterogeneity among the study effect sizes, and a fixed-effects model was applied. In contrast, *p* < 0.1 and I^2^ ≥ 50% indicated the presence of statistical heterogeneity among the study effect sizes, and a random-effects model was applied.

A forest map was used to depict the overall results of the meta-analysis. Then, a funnel plot and Egger test were used to estimate the publication bias in this study, with *p* < 0.05 indicating the presence of significant publication bias, and *p* ≥ 0.05 indicating a low risk of publication bias [49]. In asymmetric funnel plots, trim-and-fill method was used to interpolate potentially missing studies and estimate the corrected prevalence [28, 30]. Furthermore, sensitivity analysis was used to test the robustness of the results obtained in this study [50], and heterogeneity factors were identified using subgroup analysis.

## Data Availability

Data available on request from the authors.
